# Collagen Hydrolysates: A Source of Bioactive Peptides Derived from Food Sources for the Treatment of Osteoarthritis

**DOI:** 10.3390/medicines10090050

**Published:** 2023-09-01

**Authors:** Christina E. Larder, Michèle M. Iskandar, Stan Kubow

**Affiliations:** 1 School of Human Nutrition, McGill University, Ste-Anne-de-Bellevue, QC H9X 3V9, Canada; christina.larder@genacol.ca (C.E.L.); michele.iskandar@mcgill.ca (M.M.I.); 2 Corporation Genacol Canada Inc., Blainville, QC J7C 6B4, Canada

**Keywords:** peptide, collagen hydrolysate, osteoarthritis, bioavailability, human health, amino acids, nutraceuticals, supplements, digestion, first pass metabolism

## Abstract

Osteoarthritis (OA) is the most common joint disorder, with a social and financial burden that is expected to increase in the coming years. Currently, there are no effective medications to treat it. Due to limited treatment options, patients often resort to supplements, such as collagen hydrolysates (CHs). CHs are products with low molecular weight (MW) peptides, often between 3 and 6 kDa, and are a result of industrialized processed collagen. Collagen extraction is often a by-product of the meat industry, with the main source for collagen-based products being bovine, although it can also be obtained from porcine and piscine sources. CHs have demonstrated positive results in clinical trials related to joint health, such as decreased joint pain, increased mobility, and structural joint improvements. The bioactivity of CHs is primarily attributed to their bioactive peptide (BAP) content. However, there are significant knowledge gaps regarding the digestion, bioavailability, and bioactivity of CH-derived BAPs, and how different CH products compare in that regard. The present review discusses CHs and their BAP content as potential treatments for OA.

## 1. Introduction: Osteoarthritis

Osteoarthritis (OA) is the most common joint disorder, with a social and financial burden that is expected to increase [1,2]. OA is considered a heterogeneous disease that may occur in different synovial joints with various stages and degrees of severity [3,4]. The Global Burden of Disease (GBD) project, using data up until 2017, has estimated that there are over 303.1 million cases of hip and knee OA [2]. Although seemingly a large number, this GBD report is likely an underestimation, as a greater number of hand and foot cases have been diagnosed, and because of the lack of accurate data for some countries, such as Central America and sub-Saharan Africa. In fact, a commentary published in The Lancet has estimated that the global burden of OA is likely more than 500 million, or about 7% of the global population [5]. Patients with OA experience pain or aching in the joints, as well as stiffness, swelling, decreased mobility, and joint space narrowing [4,6]. As a result, reduced functionality and disability are common, as well as a significant reduction in the quality of life and mental health [3,6]. The development of OA is not caused by a singular event and may occur or progress differently between patients. For this reason, there are a vast variety of systemic and local risk factors that affect the development, progression, and stages of OA [1]. Risk factors include age, sex, ethnicity, and race [1,3], as well as poor dietary choices and reduced physical activity [4]. In addition, OA is highly associated with obesity and other metabolic conditions including diabetes, hypertension, and dyslipidemia [3,7,8,9,10], as well as an increased risk of metabolic syndrome [11,12]. Other local risk factors for OA include previous injury, occupation, and participation in contact sports [1,2,3].

### 1.1. Joint Tissues

**Figure 1:**
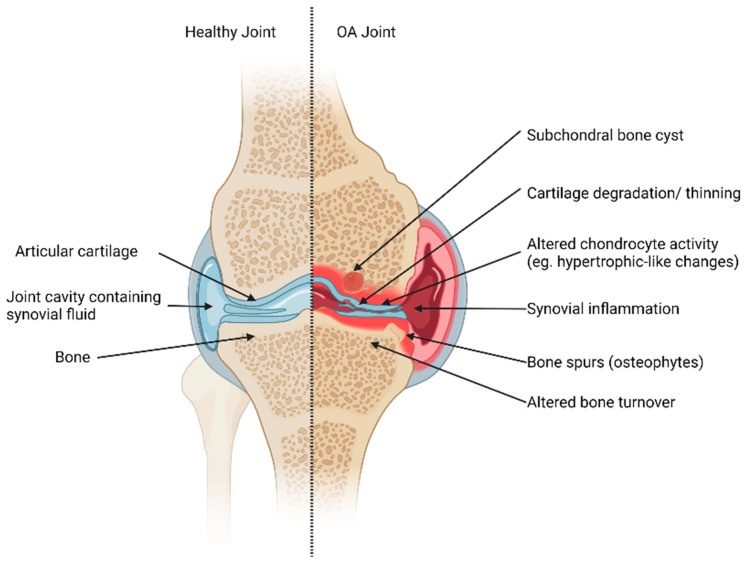
Articular structures of a synovial joint (knee joint example). Healthy tissues (**left**) and changes to joint tissues in OA (**right**). In a healthy joint, cartilage maintains its structural integrity. The subchondral bone turnover is tightly regulated, and the joint cavity has no signs of synovial inflammation. In contrast, the OA-affected joint exhibits pathogenic changes, which may include the presence of subchondral bone cysts, degeneration and thinning of cartilage, altered chondrocyte activity, synovial inflammation, and dysregulated bone turnover that may promote bone spurs. These alterations collectively contribute to the pathogenesis of OA and highlight the multifaceted nature of joint tissue changes in this condition. Created with Biorender.com.

OA has been classically defined as a “wear and tear” disease characterized by decreased cartilage [13]. Recent work, however, has helped establish that OA is a condition of the whole joint, where all articular structures form a joint and play a significant role in joint health [13]. For these reasons, it is important to understand how all the tissues present in a joint may regulate joint health. An overview of the articular structures composing a joint can be found in Figure 1.

Synovial joints consist of various tissues and components that can be affected by OA (Figure 1). These types of joints contain articular cartilage, a structure with a very low coefficient of friction, which provides a smooth surface for joint motion during movement [14]. The subchondral bone is found underneath articular cartilage and provides a supporting function as well as shock-absorbing qualities [13]. Another major joint structure is the synovium and the joint cavity containing synovial fluid. Synovial fluid is a lubricating fluid that ensures joint homeostasis and health. Other structures play supporting and regulating functions in synovial joints such as ligaments, menisci, and fat pads.

#### 1.1.1. Cartilage

Articular cartilage, also known as hyaline cartilage, is comprised of an extracellular matrix (ECM) and cells that synthesize cartilage, called chondrocytes [15]. The ECM is primarily comprised of water, which represents up to 65–80% of the total weight of healthy cartilage. The remaining components of the ECM are proteins such as collagen and proteoglycans, with a very small proportion of non-collagenous proteins, lipids, phospholipids, and glycoproteins. The main proteoglycan component of the ECM is aggrecan, which aggregates with hyaluronic acid found within joints [15]. These macromolecules are also attached to glycosaminoglycan chains, mainly chondroitin sulfate and keratan sulfate. The ECM is maintained and repaired by the resident cells, known as chondrocytes. These cells are highly specialized metabolically active cells originating from mesenchymal stem cells, yet they have very limited potential for replication [15]. For this reason, the healing capability of cartilage in response to injury, aging, and other external and internal factors is limited [15]. These cells also seldom communicate with each other for direct signal transduction via cell-to-cell contact; instead, they are sensitive to surrounding stimuli by growth factors, mechanistic load, and inflammatory signals, which can be modulated by circulating bioactive peptides [15,16].

#### 1.1.2. Bone

Bone is a highly mineralized connective tissue [13,17]. In synovial joints, the bone consists of the trabecular bone and the calcified cartilage zone. Between these layers, there is the subchondral bone plate [18]. The subchondral bone plate is permeable to small molecules and thus is one of the main routes of providing nutrients and other essential compounds to articular cartilage. The subchondral bone is a dynamic tissue that continually undergoes remodelling. Bone remodelling involves two main cell types: osteoblasts (OBs) and osteoclasts (OCs) [13]. OBs are immature bone cells that contribute to the formation of new bone, whereas OCs are large multinucleated cells that resorb bone [13].

Bone remodelling is a dynamic process that is tightly regulated by the crosstalk between OBs and OCs [13,19]. The well-established receptor activator of nuclear factor kappa-β (RANK)/receptor activator of nuclear factor kappa-B ligand (RANKL) pathway plays a key role, where OBs secrete RANKL, which binds to RANK receptors found on OC precursors. This binding induces OC differentiation and fusion, which are necessary to form mature multi-nucleated OCs. The Nuclear Factor of Activated T Cells 1 (NFATc1) is a transcription factor regulated by RANK signalling. NFATc1 upregulates the expression of tartrate-resistant acid phosphatase (TRAP) activity, a key cytochemical marker of OC function, as well as regulates cathepsin K, the main enzyme degrading collagen type I [20].

Recent research has demonstrated that, besides the RANK/RANKL pathway, co-stimulatory signals may be required for both the expression of OC-specific genes and the activation of OCs [20]. One of these co-stimulatory pathways is the activation of osteoclast-associated receptor (OSCAR). Collagens act as ligands for OSCAR, and specific sequences have been shown to interact with the receptor at different levels of affinity [20]. A recent study by Park et al. (2020) demonstrated a clear link between OA and OSCAR expression [21]. Both mouse and human cartilage show increased expression of OSCAR during OA pathogenesis. Furthermore, in OA-induced mice, the markers of OA were significantly reduced in OSCAR^−/−^ mice. The authors have suggested that disruption of OSCAR expression or the interaction between OSCAR and collagen fragments may provide an interesting opportunity for the development of therapeutic agents for OA.

Osteoblasts, like OCs, are under tight regulatory and differentiation control. Runt-related transcription factor 2 (Runx2) and transcription factor Sp7 (Osterix) play a significant and key role in OB differentiation and downstream signaling [22,23,24,25,26]. Both transcription factors regulate the expression of osteoblastogenic markers such as alkaline phosphatase (ALP) and Col1a1 [24]. Previous studies have also shown that other downstream collagen-degrading proteins such as MMP13 may also be under the control of Runx2 [27].

### 1.2. Cellular Changes in OA Joints; Inflammation

The onset and progression of OA can be characterized by multiple changes in morphology as well as cellular signalling in the joint, which can also vary between OA stages and individuals [1,13,14,28,29,30]. In recent years, inflammation has been shown to play a key role in the onset and progression of OA [14]. The regulation of chondrocyte activity, as well as bone cell activity, is directly affected by circulating levels of cytokines [14,24,31,32]. Pro-inflammatory cytokines such as interleukin-1β (IL-1β) and tumour necrosis factor-α (TNF-α) function by decreasing the synthesis of ECM components while simultaneously promoting cartilage catabolism, as well as by acting as anti-osteoblastogenic agents [24,31,32]. In some cases, the chondrocyte phenotype can change in OA [33]. As a result, ALP, Runx2, and proteins such as osteocalcin and osteopontin can be increased [33].

Recent work has helped to understand some of the macroscopic and cellular changes that occur to joints in OA; however, the exact mechanisms remain to be fully elucidated. OA is a complex and dynamic condition, where patient pathology varies greatly between individuals as well as between OA stages, types, causes, and anatomical locations. For these reasons, the choice and development of treatment options for patients remain extremely difficult.

### 1.3. OA Treatments

Although several studies have attempted to develop disease-modifying drugs (DMDs) for OA, all attempts have failed [4,34], most likely due to the heterogeneity of the disease. Current treatments are limited and often attempt to address OA symptoms such as pain rather than improve joint structure and function. These include treatment options such as transcutaneous electrical nerve stimulation (TENS) as well as pain medications. Paracetamol and non-steroidal anti-inflammatory drugs (NSAIDs) are medications often prescribed to help manage OA pain [2]. However, their continuous use is controversial and has also been shown to have a variety of side effects, such as an increased risk of abnormal liver function and detrimental gastrointestinal (GI) side effects [6,35,36,37]. Physical therapy is often advised for patients and has shown significant patient improvement outcomes, especially for knee OA, as compared to other OA treatments such as intra-articular corticosteroids (IAC) injections [2]. However, physical therapy alone cannot completely prevent, or reverse OA progression and the use of IAC injections is still controversial, as repeated use may contribute to disease progression [2]. Procedures such as autologous chondrocyte transplantation or implantation of osteochondral scaffolds are also available, but they depend on early stage OA detection, which remains a limiting factor as biomarkers are still being investigated [34].

Castañeda and Vicente (2017) remarked that antiresorptive drugs and bone-forming agents often used for osteoporosis treatment may also help prevent OA progression [13]. These therapeutic agents include estrogens, bisphosphonates, anti-RANKL-antibodies (denosumab), and strontium ranelate. However, clinical evidence is currently lacking to support their use. Surgical treatments that focus on bone include osteotomy, also known as bone realignment surgery, help to relieve OA pain. Patients with severe OA and cartilage degeneration typically undergo joint replacement surgery, but not every type of OA can be addressed by surgery [6,34]. This option is seen as a last resort due to the significant costs and risks associated with surgery, and the required post-operative care and therapy. Furthermore, many patients have also reported that they were not satisfied with their surgical intervention [38]. Regenerative medicine approaches such as gene and cell therapy to treat OA lack enough clinical evidence to become first-line options and are still being explored [34]. Further in vitro studies and clinical work on these approaches are needed.

## 2. Nutritional Supplementation: Collagen Hydrolysates

With limited treatment options and currently no approved DMDs, the potential role of nutritional factors and supplements to treat OA warrants further investigation. This is especially true for patients with pre-clinical or symptomatic OA, as early intervention is key to stopping OA progression and eventual surgery [34]. Consumers are generally interested and open to supplementation, especially if they demonstrate efficacy in promoting health improvement [39,40]. Commonly used and readily available supplements advertised to help manage OA are collagen-based products, such as gelatin and CHs [16,41].

### 2.1. Collagen

Collagen is the most prevalent protein in animals and constitutes approximately 30% of total body protein [42]. It is a structural protein found in connective tissue and characterized by the repeating motif “glycine (Gly)-X-Y”, where X is often proline (Pro) and Y is hydroxyproline (Hyp) [42,43]. Collagen has three α-chains of approximately 1000 amino acids (AAs), each of which coils around the other to form a triple helix structure. Collagen triple helices cross-link together using telopeptides found at the ends to form collagen fibrils. Several fibrils align to form collagen fibres. This cross-linking is highly conserved between collagen types. Currently, 29 types of collagen have been identified, although they can vary in amino acid (AA) sequence, structure, function, and associated distribution in tissues and organs [43]. For example, type 1 collagen is typically found in bone, skin, teeth, and tendons, whereas type II is found in cartilage [16].

### 2.2. Collagen Hydrolysates

Collagen can be isolated from various sources, including bovine, porcine, and piscine [16,44]. Collagen extraction is often a by-product of the meat industry, and the main source of collagen for collagen-based products remains bovine due to its high availability as well as biocompatibility and weak antigenicity [43]. Collagen can be extracted from various tissues such as bones, tendons, and connective tissues [16]. Marine sources of collagen include those mentioned but also skin and scales [16].

**Figure 2:**
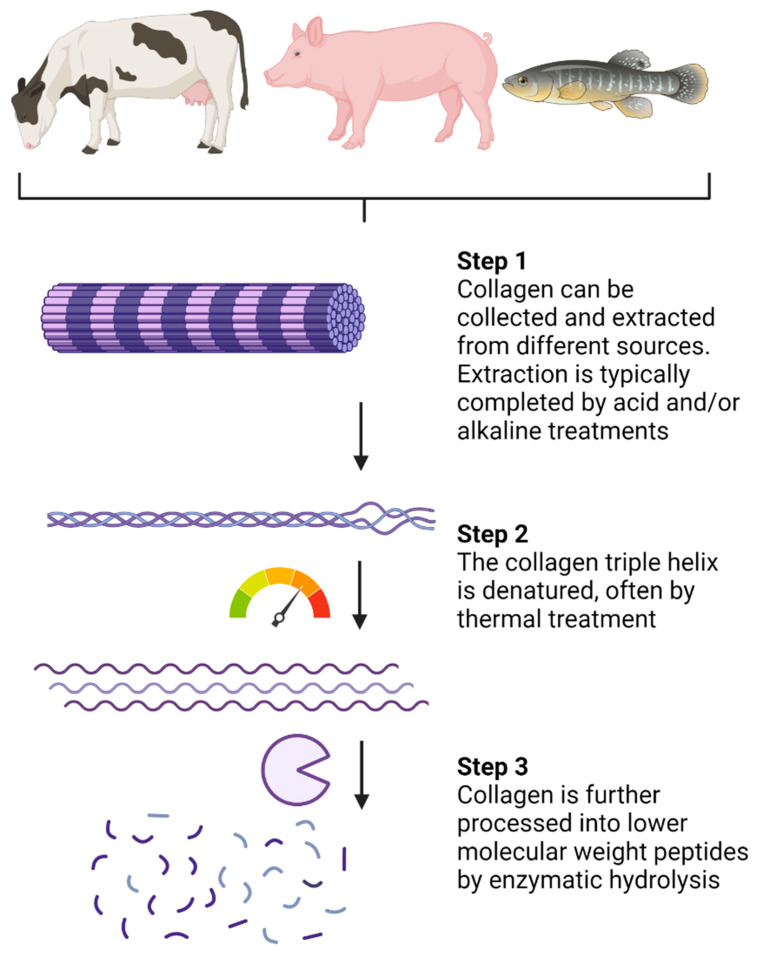
Processing of native collagen into small low-molecular-weight peptides. Created with Biorender.com.

Collagen hydrolysates (CHs) are products with low molecular weight (MW) peptides, often between 3 and 6 kDa [16]. CHs are a result of industrialized processed collagen (summarized in Figure 2) and are sold in the cosmetic, pharmaceutical, and food sectors [16]. Extraction can be completed by acid and/or alkaline treatments [16,43]. Following extraction, thermal treatments, usually above 40 °C, promote denaturation of the collagen chain triple helix (Figure 2). Enzymatic hydrolysis is then completed to break down peptide chains into lower MW peptides. The choice of processing procedure and proteolytic enzymes can vary between CH products, although pepsin, papain, and Alcalase are often used [16]. In comparison, gelatin is also a collagen-based product, but it is obtained through partial hydrolysis of collagen and is, therefore, processed to a lower extent than CHs [44]. The conversion of collagen and gelatin into bioactive products such as CHs makes collagen-sourced products valuable to the pharmaceutical and cosmetic industries.

CHs are nutraceuticals that have multiple applications and are often taken as oral supplements. The health benefits of CHs have been largely ascribed to their bioactive peptide (BAP) content and corresponding sequences [16,41,44]. The peptide content of CHs is a result of the collagen source and the processing methods described above [16]. Different processing procedures and sources will result in variable peptide sequences and content after extraction and hydrolysis, thereby affecting the overall bioactivity of the CH product. In addition to their BAP content, CHs also contain AAs which contribute to their bioactivity [16,41,45].

CHs have been demonstrated to provide several health benefits, including antimicrobial and antihypertensive effects, promotion of wound healing and bone synthesis, decreasing joint pain associated with OA, helping in the regulation of inflammation, improving skin health, acting as inhibitors of dipeptidyl peptidase-IV (DPP-IV), in addition to having antioxidant properties and angiotensin-I-converting enzyme inhibitory effects [16,41,44]. The presence of BAPs, such as Pro-Hyp, alanine (Ala)-Hyp, Pro-Hyp-Gly, and Gly-Pro-Hyp, in the blood after oral consumption of CHs and gelatin has been verified in human clinical studies [46,47,48,49]. In fact, the postprandial absorption of Gly, Pro, and Hyp was significantly greater after the oral consumption of CHs compared to non-enzymatically hydrolyzed collagen, suggesting that processed collagen products have increased absorption and bioavailability [45]. Besides their measurement in plasma, the CH-derived BAPs, Gly-Pro-Hyp, and Pro-Hyp were shown to be excreted in the urine after oral consumption, indicating that these peptides were well absorbed and stable post-absorptively [50]. Clinical studies focusing on the bioactivity of collagen BAPs have helped establish some of their health-promoting properties after oral consumption.

### 2.3. Bioactivity and Health Benefits of CHs

#### 2.3.1. Clinical Studies on CHs and CH-Derived Peptides

The clinical efficacy and safety of CHs and CH-derived peptides have been demonstrated in several trials and summarized in Table 1 [51,52,53,54,55,56,57,58,59,60,61]. The supplement primarily helps manage OA pain and increase mobility, but recent work has also demonstrated improvements in bone health and cartilage characteristics, especially in patients engaging in a physical exercise program [55,59].

A randomized double-blind, placebo-controlled study design was completed over 6 months on 250 subjects with primary knee OA to assess the efficacy of CH on OA pain and joint function [51]. Using the visual analogue scale (VAS) to assess for pain, as well as the Western Ontario and McMaster Universities Osteoarthritis (WOMAC) index pain subscale, patients showed significant improvement in knee joint pain and comfort following treatment [51]. A similar study recruited 200 patients to participate in a randomized, double-blind, placebo-controlled trial over 6 months to assess the efficacy of CH supplementation on patients with knee OA, but also included patients with hip, elbow, shoulder as well as hand and/or lumbar spine OA [53]. The number of clinical responders, as assessed using VAS, was significantly greater in the CH treatment group compared to the placebo group, and CHs were confirmed to be safe and well tolerated by patients [53]. Significant reductions in WOMAC and VAS scores were also observed in another randomized, double-blind, placebo-controlled clinical study investigating the effectiveness of porcine and bovine CH-derived peptides in patients with knee OA [54]. Although the above study provided an initial indication that the bioactive component of CHs was peptides, they were not sequenced [54]. Instead, an analysis of the AA composition of the porcine and bovine hydrolysates was completed. The composition (g/kg dry weight) was different between the two collagen sources for some AAs. For example, proline was found in greater amounts in the bovine hydrolysate (1.620) compared to porcine (1.550); however, the impact of the AAs profiles on the clinical outcomes was not discussed. The bovine hydrolysate appeared to decrease the WOMAC and VAS scores to a greater extent, but no formal analysis comparing the efficiency of the two hydrolysates was performed.

Recent clinical studies have also demonstrated that collagen peptides alongside resistance training improved body composition. A randomized double-blind placebo-controlled study showed that collagen peptides increased fat-free mass, bone mass, and muscle mass more so than the placebo [55]. The mentioned collagen peptides were part of a commercial product provided by Gelita AG (Eberbach, Germany); however, the sequences and content of the peptides were not provided nor investigated. Instead, the AA composition was assessed (no method given). In a similar study, a triple-blind placebo-controlled randomized controlled trial instructed patients with knee pain to complete a home exercise program together with a treatment of either CH or a placebo [57]. Patients treated with CH showed significant improvement in joint structures, which included decreased cartilage abrasion and lateral meniscus protrusions, as well as a significant increase in cartilage thickening in the central portion of the trochlear articular cartilage [57]. In addition, for the patients who were non-compliant with the home exercise program, VAS scoring still indicated that CH treatment decreased pain [57].

Interestingly, CH supplementation also appears to improve activity-related joint pain, regardless of OA diagnosis. For example, Clark et al. (2008) performed a 24-week clinical study involving 147 healthy athletes with activity-related joint pain who were physically active, fit, and had no evidence of established OA [58]. Joint discomfort and pain in the CH-treated group were significantly reduced. The authors suggested that CHs support joint health and could reduce the risk of further joint deterioration in high-risk groups (e.g., athletes), which merits further investigation. Thus, CHs could act as a preventive treatment and be recommended before potential OA diagnosis. Furthermore, athletes with knee pain also showed improvement in activity-related pain intensity after treatment with collagen BAPs over a 12-week supplementation period [56]. Accordingly, high-risk groups could possibly benefit from supplementation at a young age (e.g., below 25 y) and possibly delay the onset of joint damage, but this remains to be assessed. More recent work by McAlindon et al. (2011) demonstrated that, in a randomized, placebo-controlled, double-blind imaging study, changes in proteoglycan content in knee cartilage, as well as improvement in cartilage morphology, were observed after 24 weeks of CH treatment [59].

CHs have also been shown to improve other articular structures of the joint besides cartilage, most notably in the bone. In a randomized, placebo-controlled double-blind study, König et al. (2018) concluded that bone mineral density (BMD) increased after collagen peptide supplementation compared to placebo [52]. Furthermore, bone markers from plasma also showed significant improvement in bone remodelling homeostasis. Specifically, a biomarker for bone formation, the amino-terminal propeptide of type I collagen, increased in the collagen peptide treatment group, whereas no changes were observed in the placebo group. In contrast, an indicator of bone resorption, the C-telopeptide of type I collagen, increased in the placebo group, with no changes in the collagen treatment group. In summary, plasma biomarkers of bone turnover indicated that collagen peptides increased bone formation, while also decreasing the level of bone resorption.

Another study assessing the effect of CHs in pre-pubertal children concluded that partially hydrolyzed collagen, or in other words, gelatin, could improve bone remodelling during growth and development [60]. Further studies assessing the use of CHs or similar products on young healthy participants are needed, with thorough follow-ups as participants grow and age. This would help determine the preventative potential of CHs in terms of reducing the onset and severity of joint disorders.

There are very few studies that investigate the effects of CHs alone on bone health. Instead, CHs are often used in association with another treatment. For example, the effects of intramuscular injection of calcitonin were compared to calcitonin treatment along with the addition of CHs into the diet in postmenopausal women [61,62]. The effects of CHs and calcitonin together increased and prolonged the effects of the calcitonin drug treatment and a greater effect in inhibiting bone collagen breakdown was observed.

Although clinical evidence favouring CH supplementation for joint pain is growing, the mechanisms behind their activity are still not fully established, as well as how CHs compare to other protein hydrolysates. Furthermore, the BAPs profiles of the CHs and the main contributors to bioactivity are rarely investigated. Different processing procedures and sources can affect the peptide content in CHs, which may affect bioactivity. For many commercial CHs, the peptide sequences and technology used to make their respective CHs are protected, trademarked, and often patented. Further transparent evidence-based work is needed to assess the BAPs found in CH products and how they regulate joint health. In that regard, in vitro methods provide these opportunities to investigate the peptide content of CHs and their potential effects.

#### 2.3.2. In Vitro and Animal Studies on CHs and CH-Derived Peptides

Numerous studies and reviews have detailed the potential bioactivity of collagen products and CH-derived BAPs [16,41,44,63]. The bioactivity of collagen products depends on their source, processing, and bioavailability, i.e., the absorption of the bioactive compounds, such as BAPs and AAs, into the systemic circulation so they may exert their beneficial activity [16,44]. CH and CH-derived BAPs have been shown to exhibit antioxidant activity, ACE-inhibitory activity, metal chelating abilities, anti-diabetic properties, antimicrobial potential, and beneficial effects on bone and joint health [13,16,43,44,63]. The list of BAPs identified from CHs continues to grow, as well as the associated bioactive functions of the identified peptides. The efforts to create BAP databases have begun [64,65,66], although these databases remain incomplete. Other databases on proteins and BAPs stemming from CHs and others are also available, and a list of these can be found at https://biochemia.uwm.edu.pl/bioactive-peptide-databases/ (accessed on 4 August 2023). Some well-established and utilized databases are often updated, maintained, and replenished, although as with other online tools, website maintenance, broken links, offline sites, link removals, and other errors remain an issue when utilizing web-based tools and remain a limiting factor for knowledge access regarding BAPs. For the purposes of this review article, the focus will be placed on the capacity of CH and CH-derived BAPs to exert a beneficial action on cartilage, bone, and OA risk factors. For a detailed list of the other potential bioactivities of CHs and related products, reviews by Fu et al. (2019), León-López et al. (2020), and Pal et al. (2016) provide comprehensive summaries of the literature.

Previous in vitro and in vivo work by Nakatani et al. (2009) has helped establish the chondroprotective effects of porcine CHs, and its main BAP, Pro-Hyp [63]. Using an animal model, C57BL/6J mice were placed on a basic AIN-93G diet, alongside a treatment of excess phosphorus to induce joint damage. Different treatment diets included a negative control (no phosphorus) and control diet (gluten hydrolysate used as a control and excessive phosphorus), a CH treatment group (CH and excessive phosphorus), as well as a peptide group (Pro-Hyp and excessive phosphorus). Mice treated with phosphorus showed joint degradation, notably a decrease in chondrocytes and articular cartilage thickness. As a result of CH and Pro-Hyp treatment, these supplements inhibited the loss of chondrocytes induced by excess phosphorus while also inhibiting cartilage thinning [63]. In the same study, an in vitro model was used where chondrocytes (ATDC5 cells) were treated with Pro, Hyp, Gly, a combination of Pro and Hyp, a mixture of Pro, Hyp and Gly, the single peptides Pro-Hyp and Pro-Hyp-Gly, as well as the same CH used in the above animal study. Key findings from this study showed that CH and Pro-Hyp inhibited chondrocyte mineralization and terminal differentiation, as assessed by alizarin red and ALP staining, respectively. Changes to the ECM components were also observed, notably an increase in glycosaminoglycan, which was determined via alcian blue staining. Using reverse transcription-polymerase chain reaction (RT-PCR), the mRNA content of aggrecan was shown to be increased with Pro-Hyp treatment. Additionally, the expression of RunX1 and osteocalcin decreased with Pro-Hyp treatment. No RT-PCR analysis was performed on CH-treated cells. In summary, this study was one of the first to clearly establish that Pro-Hyp is a chief bioactive component associated with the observed clinical efficacy of CHs in the treatment of OA, specifically on cartilage tissue [63].

Another BAP derived from CHs is Gly-Pro-Hyp, which has been suggested to be involved in platelet aggregation, which was recognized by platelet glycoprotein VI [67,68]. This interaction is unique; Gly-Pro-Hyp occurs rarely in other proteins, except for collagen, and glycoprotein VI is thought to be expressed solely by platelets. Furthermore, this tripeptide, which was generated by hydrolyzing porcine, bovine, fish, and chicken collagen with *Streptomyces* collagenase has been shown to inhibit the activity of DPP-IV, which has been associated with diabetes [68]. The production of Gly-Pro-Hyp depended on the source of the collagen. Porcine and bovine collagen yielded greater Gly-Pro-Hyp content compared to fish and chicken collagen after hydrolysis. Other generated peptides (Gly-Ala-Hyp and Gly-Pro-Ala) were assessed for their DDP-IV activity, although only Gly-Pro-Hyp showed bioactivity. This peptide might prove to be an important health modulator in OA as patients diagnosed with diabetes are at an increased risk of developing arthritis [10,69,70].

Preliminary in vitro studies using bone marrow macrophages differentiated into OCs showed that collagen decreased the number of differentiated OCs, i.e., positively tartrate-resistant acid phosphatase (TRAP) stained cells. Other reports have shown that CHs from animal skins decreased OC resorption area but did not affect OC growth [71]. A more recent study by N’deh et al. (2020) used OC precursor RAW 264.7 cells and demonstrated that collagen extract from chicken decreased the mRNA levels of TRAP and cathepsin k [72]. Collagen from Yeonsan Ogye chicken flesh was extracted in a pressure chamber at 121 °C, 0.5 mPa for 30 min, filtered, and then lyophilized until used for the study.

The effects of CHs on OBs have been more thoroughly investigated, although the mechanisms of action remain to be fully established. Previous work using bovine collagen on a pre-osteoblast cell line (MC3T3-E1 cells) observed changes in gene expression, primarily an increase in Runx2 [22]. The increased expression of ALP activity and mineralization was also observed. Another in vitro study using MC3T3-E1 cells helped establish that the BAP and Pro-Hyp promoted osteoblastic differentiation, but not proliferation [73]. Pro-Hyp treatment was shown to upregulate both osteoblastic differentiation genes Runx2 and Osterix, as well as Col1a1. The application of the collagen tri-peptide Gly-Pro-Hyp on MC3T3-E1 cells also showed upregulated protein expression of Runx2, Osterix, ALP, and Col1a1 in a dose-dependent manner [23]. Using the human osteoclastic MG-63 cells, CHs were shown to stimulate ALP activity, calcium deposition, and collagen synthesis [74]. The CH was porcine-sourced and hydrolyzed with a combination of protamex + flavorzyme at 50 °C for 12 h, fractioned and filtered (<3 kDa), and freeze-dried.

## 3. Digestion and Bioavailability of CHs and CH-Derived Peptides

### 3.1. Gastrointestinal Digestion

When consumed, CHs, BAPs, and any other nutraceuticals or medications taken orally must undergo digestion and absorption before exerting their bioactive effects [17,75]. It is mainly in the small intestine (SI) that proteins are broken down into peptide and AA components. However, the final breakdown of proteins and larger peptides to their smaller components occurs on the surface of resident intestinal enterocytes by brush border enzymes. The BAPs released after protein and peptide digestion are absorbed by villus enterocytes [76]. The remaining food components and nutrients that are not absorbed in the SI travel to the large intestine. In these colonic regions, non-dietary carbohydrates, proteins, peptides, and AAs can be fermented by resident microbiota [77,78]. These fermented food compounds are also known as prebiotics, which play an important role in our health.

### 3.2. Absorption and Hepatic First Pass: Bioavailability of CHs and CH-Derived BAPS

**Figure 3:**
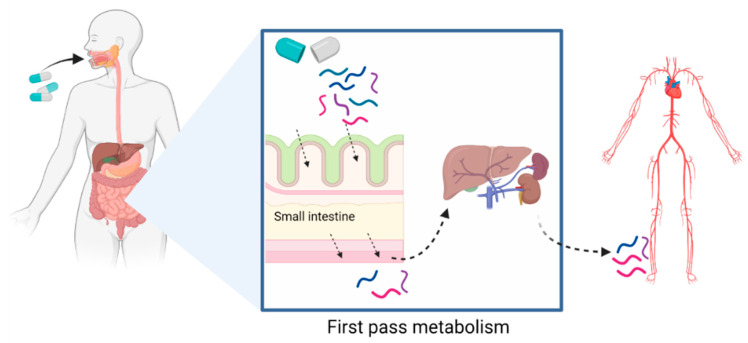
First-pass metabolism often applies to orally administered drugs or nutraceuticals and is the process defined by the absorption of metabolic compounds at the level of the intestinal epithelium, followed by hepatic metabolism, before being released into the blood. Created with Biorender.com.

After digestion, BAPs undergo first-pass metabolism, which mediates the entry of bioactive molecules into the systemic circulation [79,80]. This process is defined by the absorption of metabolic compounds at the level of the intestinal epithelium, followed by hepatic metabolism, before being released into the blood (Figure 3). The additional release of BAPs after CH digestion may occur, as the CH peptide components can be broken down into smaller BAPs and AAs in the stomach and SI. Regardless of the extent of digestion, the bioactivity of CH-derived BAPs, and therefore clinical efficacy, depends heavily on their bioavailability, which is the proportion that reaches the systemic circulation unaltered upon oral ingestion [75,79]. Peptide bioavailability remains one of the greatest factors affecting bio-potency, and the digestion and absorption of different CHs can differ greatly, which may lead to altered CH bioactivities [44,81].

**Figure 4:**
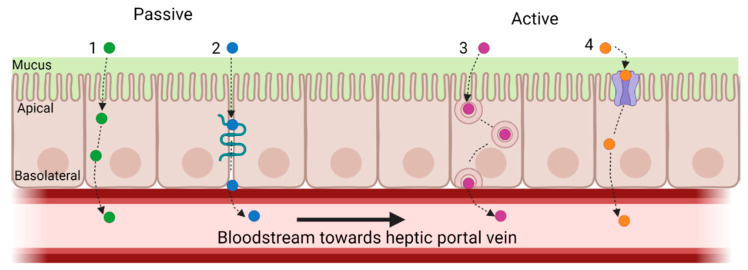
Main routes of peptide absorption by intestinal enterocytes: (1) passive diffusion; (2) paracellular transport; (3) transcytosis; and (4) carrier-mediated transport. An example of carrier-mediated transport is peptide transporter 1 (PepT1), which is a high-capacity, low-affinity, proton-coupled transporter that facilitates the movement of peptides from the gastrointestinal lumen into the intestinal epithelium. PepT1 is largely responsible for the transport of di- and tri-peptides, especially those with low MWs. Created with Biorender.com.

Large MW peptides are less effectively absorbed than lower MW peptides, so CHs with lower MWs are more likely to be absorbed to exert their bioactivity [16,46]. There are four main routes of peptide absorption by intestinal enterocytes: (1) passive diffusion; (2) paracellular transport; (3) transcytosis; and (4) carrier-mediated transport (active transport) (Figure 4) [75].

Peptides that pass through the intestinal layer are typically transported by diffusion or active transport. In passive transport, molecules such as peptides may pass through the apical and basolateral membranes of the intestinal epithelium without expending energy. This type of transport depends on the properties of the peptides, such as size (MW and chain length), charge, and hydrophobicity, all of which depend on AA content [16,75]. Paracellular peptide transport involves passive absorption via the tight junction gaps between epithelial cells [75]. Tight junctions act as barriers that prevent the passage of substances through the intercellular space between adjacent cell membranes, thereby restricting the penetration of polar macromolecules. Only 0.01–0.1% of the total intestinal surface area consists of water-filled pores/channels found between cells. As such, there is limited peptide permeability via this pathway [75]. Transcytosis is an energy-driven process that involves the transportation of substances across polarized cells, such as intestinal epithelial cells [75]. Substances are transported across cells in internalized vesicles called endosomes. Given that peptides need to interact with the apical lipid bilayer via hydrophobic interactions before being internalized, longer-chain hydrophobic peptides are more likely to be absorbed by transcytosis. For example, the bioactive peptide YWDHNNPQIR is thought to be transported by transcytosis due it its hydrophobic amino acid content. Carrier-mediated transport is an active type of transport, as the movement of molecules, nutrients, and peptides is against their concentration gradient. Intestinal peptide transporters utilize H + gradients to provide an uptake of single amino acids and di- and tri-peptides into the brush border epithelial cells [75]. Peptide transporters 1 (PepT1) and 2 (PepT2) are part of the solute carrier (SLC) family. Pept1, also known as SLC15A1, is present on the apical side of the small intestinal epithelial cells. It is a high-capacity, low-affinity, proton-coupled transporter that moves peptides from the GI lumen into the intestinal epithelium [75]. The transport of di- and tri-peptides, especially with low MW, is mainly due to the activity of PepT1 [75].

### 3.3. In Vitro Models of Digestion and Absorption

#### 3.3.1. Digestion

Bioaccessibility pertains to the fraction of food, molecule, or compound that is released from the food matrix during digestion and is, therefore, available for absorption via the intestine [82]. Ideally, in vivo studies using humans provide the best evidence for measuring bioaccessibility; however, human studies are costly, lengthy, have small sample sizes, and are restricted by ethical parameters [83,84]. Animal studies are another alternative, but may not always reflect human studies of nutrients and bioactive food components due to differences in metabolic activity and digestive enzymes between animals and humans [75,84,85].

Instead, in vitro models of digestion provide a rapid, cost-effective, and simple method to assess bioaccessibility [82,83,84,85]. These models can be highly controlled, more so than animal or human studies, making them highly reproducible while providing multiple sampling options. A major advantage of using such in vitro models is that they allow for high-throughput screening of the effects of digestion on nutrients, or nutraceuticals such as CHs, to determine the BAPs that are released and those that resist digestion. This tool also allows for an initial screening among different CH products to determine potential differences in bioactivity stemming from the different BAP profiles released following digestion. In vitro models thus provide a platform for completing preclinical digestion studies to identify bioactive compounds or molecules such as BAPs that are generated by digestive processes, which could become bioavailable. Although in vitro models are useful, several digestion protocols have been published using different experimental conditions, thus comparing results often become difficult [75]. For this reason, an international consensus on the digestion conditions was reached within the European COST Action Infogest (http://www.cost-infogest.eu/ (accessed on 4 August 2023)). The usability of this standardized approach is growing.

Besides in vitro studies, in silico models could provide an additional platform for methodological approaches investigating BAPs. In silico methods have previously been used to identify BAPS from protein sources such as cereal, milk, and potato proteins but have been underutilized to identify BAPs from CHs [44]. Advances in quantitative structure-activity relationship and molecular docking models are ongoing and have been previously used to help understand the binding of some collagen peptides to angiotensin-converting enzyme (ACE). Nevertheless, improvements among in silico prediction models are still needed, as they do not always reflect in vitro or in vivo results and often ignore post-translational and AA modifications.

Chen et al. (2020) utilized a simulated in vitro digestion model to determine the effect of different MW peptide fractions from tilapia skin collagen on zinc chelation capacity and bioaccessibility [86]. Pepsin and pancreatin were used to simulate the gastric and intestinal digestive phases, respectively. The results demonstrated that zinc bioaccessibility was improved by the low MW collagen-derived peptides with strong zinc chelating abilities [86]. Such studies demonstrate the utility of in vitro tools assessing the digestibility of food components and nutraceuticals before costly animal and clinical studies are undertaken. Another study demonstrated that BAPs were generated after in vitro digestion of Alaska pollock skin CH [87]. Notably, the copper-chelating activity of this CH was significantly increased after simulated digestion, which suggested that digestion contributes to the increased bioactive potential of CHs. Another study investigating marine skin collagen identified BAPs with ACE-inhibitory capacity after in vitro digestion [88]. Collagenous residues from squid skins were processed and fractioned and then digested using pepsin and pancreatin to simulate upper intestinal digestion. Pepsin had almost no effect on the MW of the processed peptide fractions, whereas the digestive action of pepsin and pancreatin resulted in an increase in lower MW peptides and increased ACE-inhibitory capacity. Therefore, upper intestinal digestion may increase the bioactivity of CHs and remains a useful screening tool before in vivo studies are completed. Also, using liquid chromatography-isoelectric focusing and tandem mass spectrometry, the decapeptide Gly-Arg-Gly-Ser-Val-Pro-Ala-Hyp-Gly-Pro was identified after the digestion of squid skin collagen and demonstrated high ACE inhibitory bioactivity [88]. The latter bioactive peptide could contribute to the overall health-promoting capacity of CHs, but targeted methods of quantification of this peptide are needed, as well as bioavailability assessments.

Although small MW peptide content significantly contributes to CH bioactivity, no methods of targeted peptide analysis have been described in the literature for CH digesta. Previous methodological approaches, developed for plasma samples, often calculated peptide content using indirect calculations of Hyp-containing peptides and/or AAs [16,45,46,47,48]. In response to the need for targeted peptide quantification for CH digesta, we utilized a standard in vitro model of upper intestinal digestion and developed a rapid and sensitive method of analysis using capillary electrophoresis for BAPs after in vitro digestion [89]. The concurrent analysis of the free AA content, as they may also contribute to CH bioactivity, was determined using LC-MS equipped with a hydrophilic interaction chromatography (HILIC)-Z column. Digestion efficiency and the release of different peptide sequences are a direct result of differing CH preparation or purification methods to ensure that CHs are more digestible and can impact peptide profile, bioavailability, and downstream bioactivity [77,90,91].

Previous studies, such as the study completed by Simons et al. (2018) investigating CH composition and bioactivity, have often failed to account for digestive processes [81]. A recent review by Amigo et al. (2020) also noted that cell-based experiments used to investigate the bioactivity of food peptides are often not consistent with in vivo data, principally because in vitro studies do not consider the digestive and metabolic processes that occur before BAPs reach their target tissues [75]. Previous studies by our group have also shown that digestion affected peptide profiles and increased the number of released sequences [77]. Our work demonstrates the necessity of digesting CH products prior to subsequent analysis of bioactivity, as the peptide profiles may change significantly after digestion.

#### 3.3.2. Absorption and First-Pass Metabolism

Although screening for bioaccessibility of bioactives is key to understanding the impact of digestive processes on nutraceuticals such as CHs, the bioavailability of the released bioactive components also needs to be evaluated, which is dependent upon first-pass metabolism. First-pass metabolism is a process defined by the hepatic metabolism of compounds following their absorption at the level of the intestinal epithelium, which mediates entry into the systemic circulation [79,80]. Human clinical studies have shown that BAPs and AAs generated from orally ingested CHs and gelatin appear in the systemic circulation and are excreted in urine [45,46,47,48,49,50,92]. These bioavailable BAPs and AAs have also been shown to build up in joint tissues such as cartilage and bone [17,93,94,95], which likely explains why CHs demonstrate possible clinical potential. As with bioaccessibility measurements, the assessment of CH and peptide bioavailability using human trials continues to be lengthy, costly, and with restricted experimental opportunities for sampling due to ethical considerations, as well as limited methodologies for identifying and detecting both peptides and AAs in plasma or blood. As an alternative, animal models have also been utilized to assess BAP bioavailability from collagen and collagen precursor products [49,96,97,98], but these studies are also generally slow and costly, and the predictions of bio-absorbability do not always align with human clinical data due to species differences in intestinal permeability as well as metabolic activity [75,85]. For these reasons, cell culture models have often been used instead to investigate BAP intestinal transport [75].

The human colonic adenocarcinoma cell line, Caco-2, has frequently been used to assess the SI absorption of drugs, nutrients, and dietary components, as well as CH and CH-derived peptides [76,91,98,99,100]. The bioavailability of CH peptides typically depended on the hydrolysis method used to generate the CHs [90]. The processing of collagen to CHs is a key step in determining bioavailability; therefore, potential bioactivity remains one of the most highly investigated characteristics of CHs.

Peptide transport across the intestinal layer via paracellular pathways is primarily dependent on the charge and molecular size of the compound. A study by Sontakke et al. (2016) showed that the apparent permeability of Gly-Pro-Hyp was greater than that of Pro-Hyp [98]. As both of these peptides are uncharged, it is possible that active transporters were implicated in the relatively higher transport of Gly-Pro-Hyp. For this reason, the choice of the intestinal cell line used to predict peptide bioavailability is important, especially considering the expression and activity of active peptide transporters. Although the Caco-2 cell line is considered to be the standard to assess intestinal absorption of molecules, it is not the ideal model to investigate peptide bioavailability as PepT1 is under-expressed in these tumorigenic cells [101]. In fact, depending on the compound under investigation, especially for peptides, permeability results using this cell culture model are not always in agreement with human intestinal permeability [85,101]. Alternatively, the non-tumorigenic, human small intestinal epithelial crypt (HIEC) cell line can be utilized to circumvent the limited expression of PepT1 in the Caco-2 cell line. As HIEC cells more accurately approximate small intestinal physiological in vivo conditions, they have been demonstrated to be a better alternative to Caco-2 cells for predicting transporter-mediated absorption of orally ingested compounds in humans [102,103,104,105]. There are also advanced Caco-2 clones enriched in PepT1 that have been utilized [106].

A large limitation of current in vitro studies assessing the bioavailability of BAPs is the use of intestinal cell cultures only, without taking into consideration the hepatic effects on the BAPs following intestinal transport. Previous studies have utilized a Caco-2/HepG2 co-culture model of first-pass metabolism to assess the bioavailability of dietary components, using digesta from a human-simulated in vitro gastrointestinal digestion [80]. In vitro models such as this correlate well with data from human and animal models in terms of assessing the oral bioavailability of compounds such as xenobiotics [107,108]. It has been shown that PepT1 expression can be enhanced by Pro-Gly in HepG2 cells [109], although the hepatic effects on Pro-Gly have not been assessed. There is generally a major knowledge gap regarding the hepatic first-pass effects on BAPs after intestinal absorption.

Recent studies by our group have assessed the bioavailability of CH-derived BAPs utilizing a HIEC-6/HepG2 co-culture to more accurately simulate the in vivo conditions of absorption, as well as assess the first-pass effects of BAPs [91]. Peptide transport, first-pass effects, and bioavailability between the two different bovine-sourced CHs were determined via the measurement of BAPs (Gly-Pro, Hyp-Gly, Ala-Hyp, Pro-Hyp, Gly-Pro-Hyp) via CE. Overall, a high degree of transport was observed. For example, over 80% of Hyp-Gly was absorbed with one of the CHs assessed. There was also a notable and CH-dependent hepatic production of Ala-Hyp, Pro-Hyp, and Gly-Pro. Our study supports previous literature that has shown CHs to be well-absorbed in animal and human studies. A main feature and benefit of CH products is their bioavailability and tolerability by patients [16,44,53]. As a consequence of increased bioavailability, a greater amount and number of BAPs in the bloodstream may reach OA joints and exert their bioactivity.

## 4. Microbial Effects of Non-Digested and Unabsorbed CH Components

**Figure 5:**
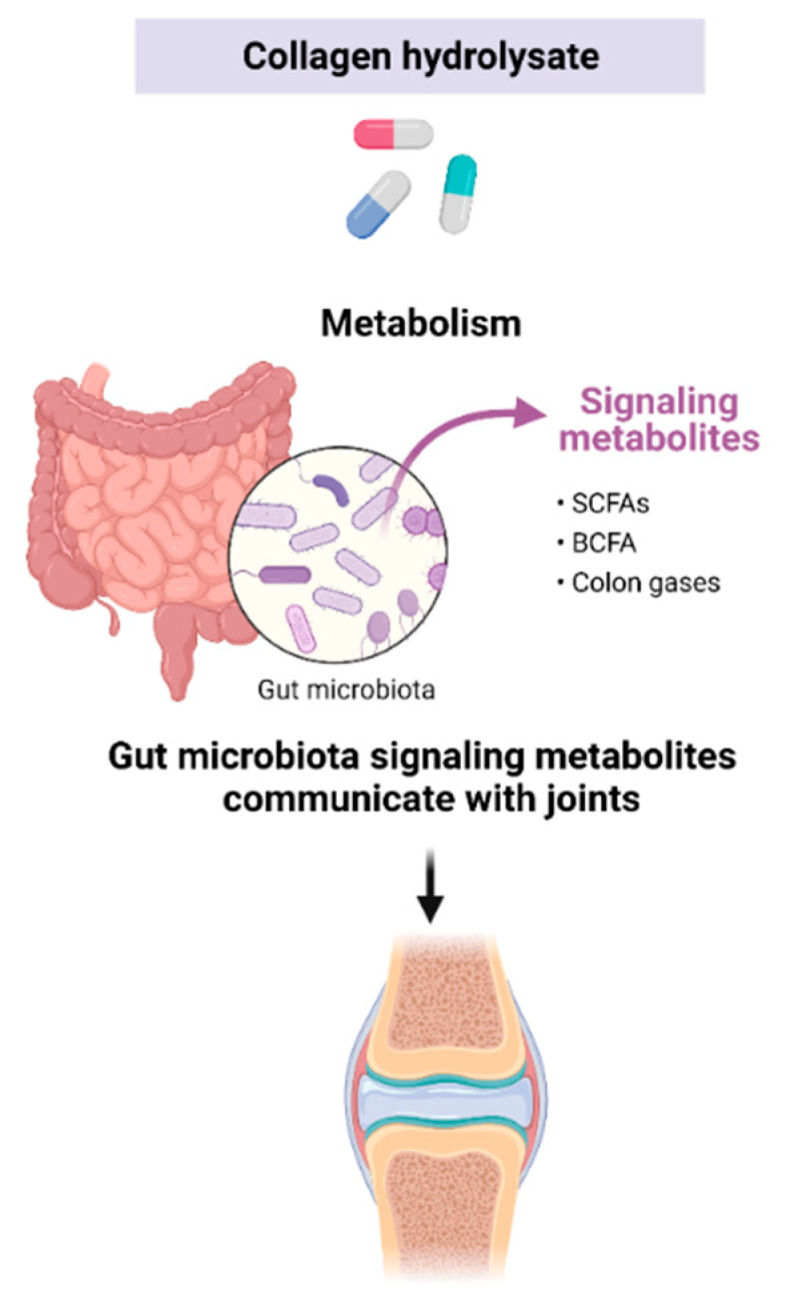
CHs may act as prebiotics and produce microbial metabolites. Colonic fermentation products may impact joint health. Created with Biorender.com.

The GI microbiome refers to the community of organisms living within the gut. This collection of microbes can be both beneficial and harmful to the host. Prebiotics are dietary components, such as nondigested carbohydrates, proteins, peptides, and AAs, which can exert favorable alterations in the behavior, growth, or profiles of the GI microbiota [78]. Prebiotics are fermented by beneficial microorganisms in the GI tract and produce short-chain fatty acids (SCFAs) such as acetate, butyrate, and pyruvate. Prebiotic microbial fermentation products have been found to offer various health benefits to the host. Prebiotics have been shown to modulate inflammation, improve conditions such as inflammatory bowel disease, provide protection against colon cancer, exhibit antioxidant activity, as well as reduce symptoms associated with metabolic disorders, including arthritis [110,111,112]. Microbial metabolites such as SCFAs and some branched-chain fatty acids (BCFAs) have also been shown to provide significant health benefits including anti-inflammatory and antitumorigenic activities. Small intestinal protein digestion can result in peptides that are able to bypass intestinal absorption and reach the colon to undergo fermentation by colonic bacteria [113]. Consequently, it is conceivable that the diverse range of peptides and amino acids found in CHs may function as prebiotics, promoting the production of microbial nitrogenous products through colonic fermentation, such as SCFAs, BCFAs, and GI gases (Figure 5). In that regard, investigating the prebiotic effects of CHs could prove significant for OA as there is a growing connection between gut health and joint health.

Interest in the vast effects that the gut microbiome and microbial metabolites have on human health has increased our understanding of the complexity of this system, but only recently has the link between the microbiome and OA begun to be recognized [30,110]. In that regard, the effect of CHs on the fermentative activities of gut microbiota was investigated by our group [77]. A dynamic GI digestion model containing human fecal matter was used. The model consists of five bioreactor vessels: stomach, small intestine, ascending colon, transverse colon, and descending colon, and each vessel is monitored and automatically adjusted for pH. The study provided the first evidence that CHs can lead to the generation of SCFAs and BCFAs with decreased levels of NH_4_, all of which are well-known biomarkers of GI health. Microbial metabolic production appeared to be dependent on the CH tested, which likely corresponds to differing peptide diversities. The study suggests that CHs may induce prebiotic effects, although only in the ascending colon. Further research is necessary to explore the biological importance of colonic metabolites derived from CHs, particularly in light of recent findings that suggest a connection between the gut and OA. As more evidence supporting the link between the microbiome and OA is gathered, the impact of CHs and their potential prebiotic effects, as mediated by their peptide and AA content, warrants further investigation.

## 5. Future Trends

Despite the growing evidence supporting the use of CHs to treat OA, their clinical efficacy remains highly speculative for many researchers. This is primarily due to the following: (1) the limited number of studies completed; (2) small patient sample sizes; (3) limited publications in reputable journals; and (4) limited treatment durations, with no long-term follow-up with patients. In addition to some promising studies establishing the positive health benefits of CHs, the literature also contains a significant portion of poorly designed and executed clinical studies, which decreases the credibility of CHs as a promising and potential therapeutic agent. As such, independent, high-quality, long-term studies are needed and recommended, so that the therapeutic properties of CHs in both younger and older populations can be assessed. Likewise, particular emphasis and critical investigation into the industrial processing of CHs and the resulting different peptide formulations, specifically BAP and AA content is needed, as patient and clinical outcomes could be affected [114]. An analysis of the peptide composition of different CHs was completed by Simons et al., (2018) [81]. Two different commercial preparations of bovine CHs (Peptan B2000 from Rousselot and CH-Alpha from Quiris Healthcare), as well as a porcine CH (Mobiforte from Astrid Twardy) were compared. MALDI-TOF results showed extensive differences between the peptide profiles of the different CHs. Some peptide peaks were unique, whereas others were common among the CHs. In one analysis batch, the two bovine-sourced CHs had only 16 common peptide peaks, with 106 and 82 unique peptide peaks for Peptan B2000 and CH-Alpha, respectively. These results highlight the impact of processing on the peptide content among same-source collagen material. Differences in bioactivity were also observed among the CHs. One batch of Peptan B200 stimulated IL-8 and MMP-1 activity in fibroblast-like synoviocytes, whereas the other bovine CH (CH-Alpha) and porcine CH showed no effect.

In silico methods remain an underutilized tool for predicting CH-derived BAP. Future studies on improving and developing in silico models to better predict BAP digestion and transport could provide an additional platform to the methodological approaches used to investigate peptide bioavailability before cell culture studies are completed. Using in silico methods may also improve predictions of digestibility and the impact of chemical modifications that affect peptide half-life, such as cyclization [75]. Encapsulation and peptide structure modification are techniques that may be used to improve CH-derived BAPS bioavailability but require more high-quality studies to determine their safety and effectiveness.

Investigations into the synergistic potential of the combined presence of BAPs and AAs in CHs are also required but have gathered little attention. Further well-designed mechanistic studies are needed to help establish the biological plausibility of BAPs and AA content contributing to joint health.

## 6. Conclusions

The health-promoting properties of CHs have been mainly attributed to their BAP content. Patients with conditions such as OA, which have limited treatment options, are generally open to using nutraceuticals such as CHs. In that regard, it is important to assess the safety, bioaccessibility, bioavailability, and bioactivity of CHs and their peptide diversity and content. The BAP content of CHs provides an interesting opportunity to use otherwise wasted by-products of the meat and fish industry. However, the mechanisms in which CH-derived BAPs support joint health need further studies to comprehensively understand their physiological impact considering their availability and general consumption.

## Figures and Tables

**Table 1:** Summary of human clinical studies using CHs to treat OA and related joint problems.

Study Design	Population	Supplement Used and Details	Reference
**Randomized double-blind, placebo-controlled**	Knee OA	Colnatur by Ordesa (Eberbach, Germany) CH with a mean molecular weight of 3500 Da. Sourced from “traceable non-ruminant bones of neutral taste and odour” Improvement in knee joint pain	Benito-Ruiz et al., (2009) [51]
**Single-center, prospective, randomized, double-blind, placebo-controlled**	Postmenopausal women with reduced bone mineral density	FORTIBONE^®^ by Gelita Described as a mixture of specific bioactive collagen peptides (SCP) with a mean molecular weight of ~5 kDa. However, peptides were not given. Fortibone is derived from Type I and III bovine collagen Increased bone mineral density	König et al., (2018) [52]
**Randomized, double-blind, placebo-controlled**	Knee, hip, elbow, shoulder, hand, and/or lumbar spine OA	Genacol AminoLock Collagen Source: Bovine collagen. No additional details in the manuscript. Reduced VAS scores	Bruyère et al., (2012) [53]
**Randomized, double-blind, placebo-controlled**	Knee OA	Porcine (supplied by NittaGelatin Inc., Osaka, Japan) and bovine (Nitta Gelatin India Ltd., Panampilly Nagar, India) CH-derived peptides. Peptide sequences not given. Reduced WOMAC and VAS scores	Kumar et al., (2015) [54]
**Randomized, double-blind, placebo-controlled study**	Elderly sarcopenic men	BODYBALANCE by Gelita using bovine type 1 collagen. Increased fat-free mass, bone mass, and muscle mass	Zdzieblik et al., (2015) [55]
**Monocentric, prospective, randomized, double-blind,** **placebo-controlled**	Athletes with knee pain	FORTIGEL by Gelita; described as a mixture of collagen peptides. Sequences not given. Decreased activity-related pain intensity	Zdzieblik et al., (2017) [56]
**Triple-blind, placebo-controlled, randomized controlled trial**	Knee pain	Genacol AminoLock Collagen Source: Bovine collagen. No additional details in the manuscript. Improvement in various joint structures	Feliciano et al., (2017) [57]
**Prospective, randomized,** **placebo-controlled, double-blind study**	Athletes with activity-related joint pain	CH-Alpha from Gelita. No details given. Diminished joint discomfort and pain	Clark et al., (2008) [58]
**Single-center, prospective, randomized, placebo-controlled, double-blind, pilot trial**	Mild knee OA	FORTIGEL by Gelita; described as a mixture of collagen peptides. Sequences not given. Increased proteoglycan content in knee cartilage and improved cartilage morphology	McAlindon et al., (2011) [59]
**Randomized double-blind study**	Pre-pubertal Spanish children	Gelatine Royal (Kraft Foods Europe, Barcelona, Spain), unspecified collagen source. Improved bone remodelling during growth	Martin-Bautista et al., (2011) [60]

## Data Availability

Not applicable.
